# How to Interpret Quality of Life Assessment of Patients With Chronic Wounds Using the Wound‐QoL

**DOI:** 10.1111/iwj.70757

**Published:** 2025-09-02

**Authors:** Toni Maria Janke, Matthias Augustin, Franziska Zirkenbach, Christine Blome

**Affiliations:** ^1^ Institute for Health Services Research in Dermatology and Nursing University Medical Center Hamburg‐Eppendorf Hamburg Germany

**Keywords:** HRQoL, interpretation, patient‐reported outcome measure, PROMs, quality of life, wound‐QoL

## Abstract

The Wound‐QoL assesses patients' health‐related quality of life. Quick and valid interpretation of the results is crucial, but no thresholds have yet been established. Additionally, counting top box responses might be a quick approximation to the Wound‐QoL score itself. The aim of this study was to develop Wound‐QoL bands (i.e., thresholds) and to analyse top box responses. Patients from European countries completed the Wound‐QoL and a global question. We grouped patients' Wound‐QoL scores and mapped these on the global question score. Upon this, we developed sets of Wound‐QoL bands and calculated the weighted kappa (κ) coefficient of agreement for each set. Moreover, we analysed the correlation of the sum of top box responses with patients' Wound‐QoL. The 305 patients (mean age: 68.5 years; 52.8% male) had most frequently leg ulcers (49.2%). The final set of Wound‐QoL bands with the highest κ coefficient (0.564 and 0.550) was 0–0.25, not at all/rarely impaired; > 0.25 to 1, a little; > 1 to 2, moderately; > 2 to 3, quite a lot; > 3 to 4, very much. Top box responses showed strong correlation with the Wound‐QoL scores (0.961–0.961). We are confident that the Wound‐QoL bands will facilitate interpretation of Wound‐QoL data in routine care as well as in research.

1

**Summary**

- This study presents newly developed Wound‐QoL bands, which are thresholds to interpret patients' Wound‐QoL scores.
- The final set of Wound‐QoL bands was as follows: 0–0.25, not at all/rarely impaired; > 0.25 to 1, a little; > 1 to 2, moderately; > 2 to 3, quite a lot; > 3 to 4, very much.
- The final set of Wound‐QoL bands show moderate agreement with the Wound‐QoL scores.
- Counting the number of items being answered on the two highest response options (top box count) can be considered a good approximation to the actual Wound‐QoL score, showing strong correlations.

## 2 Introduction

Chronic wounds are defined as ulcers that do not heal adequately over time, often due to underlying conditions or risk factors such as venous insufficiency, diabetes, or constant pressure [1]. A systematic review shows a global prevalence of approximately 1.67 per 1000 people with any type of chronic wounds [2]. The elderly have an increased risk of developing chronic wounds [3]. Chronic wounds impose a considerable burden on patients, manifesting in physical symptoms like pain, exudate, and odour, along with psychological burden including anxiety and depression [3, 4, 5]. Moreover, they can lead to financial strain as well as restrictions in daily activities and social participation [6, 7].

The subjective and multidimensional construct of health‐related quality of life (HRQoL) captures these different impairments, including bodily, emotional, mental, social, and functional aspects of life [8]. Assessing HRQoL is critical in both clinical practice and research as it supports patient‐centred care, informs treatment decisions, and monitors treatment progress [9]. Regulatory authorities recognise HRQoL as an essential endpoint in evaluating treatment efficacy [10, 11]. This emphasises the need for practical, user‐friendly, and valid HRQoL questionnaires in both routine care and research.

Therefore, the Wound‐QoL questionnaire [6] has been developed based on three longer previously existing questionnaires [12, 13, 14] for the use in patients with any type of chronic wounds. In its original version, the Wound‐QoL contains 17 items (Wound‐QoL‐17). Later, a shortened 14‐item version has been developed (Wound‐QoL‐14), in which three items were excluded [15]. The questionnaires are answered on a 5‐point Likert scale (ranging from 0 = ‘not at all’ to 4 = ‘very’). For patients completing at least 75% of the items, a total score can be calculated. In the same way, three subdomain scores can be calculated: body (5 items in the Wound‐QoL‐17, 4 items in the Wound‐QoL‐14), psyche (5 and 4 items, respectively) and everyday life (6 and 5 items, respectively). In both questionnaires, one item each is not assigned to a subdomain (item on ‘financial burden’ in the Wound‐QoL‐17; item on ‘treatment burden’ in the Wound‐QoL‐14). Both questionnaires show good performances regarding validity and reliability [16, 17, 18].

The Wound‐QoL is used worldwide in over 30 languages as a standard tool for assessing HRQoL in patients with chronic wounds and shows favourable psychometric properties (reliability and validity) in the German original as well as in other language versions (e.g., [18, 19, 20, 21, 22, 23]). Quick and valid interpretation of the results is particularly important in clinical practice. For this, the minimal important difference (MID) has been estimated to be 0.50, meaning that this is the change in HRQoL that patients would consider meaningful [24]. However, no thresholds have yet been established that help to interpret a patient's Wound‐QoL score at a given time point. Above this, analysis in clinical practice could be facilitated when clinicians concentrate on the items in which patients report the highest impairments, as this might be a quick and easy approximation of the Wound‐QoL score itself.

Therefore, the aim of this study was to develop a categorisation (i.e., Wound‐QoL bands) that allows the interpretation of Wound‐QoL data. Additionally, we aimed to analyse the value of an interpretation based on a simple count of the number of items in the Wound‐QoL responded with the two highest response options “quite” and “very” (i.e., top box responses).

## 3 Material and Methods

### 3.1 Study Population

This is a retrospective analysis using data from a European study [16], which has been approved by the local ethics committees. Inclusion criteria were being 18 years or older, having at least one chronic wound and being able to understand and complete the questionnaire. Patients were recruited in ambulatory clinics in eight countries (Austria, Lithuania, the Netherlands, Poland, Slovakia, Spain, Switzerland, and Ukraine) by the treating healthcare personal. The patients completed a paper‐based questionnaire in the respective local language. We collected sociodemographic and clinical as well as HRQoL data. As an anchor variable, patients answered a single‐item global question (GQ) to assess their overall wound‐specific HRQoL asking “How much does your chronic wound currently affect your quality of life?” on a 5‐point verbal rating scale (ranging from 0 = ‘not at all/rarely’ to 4 = ‘very much’). To analyse the Wound‐QoL scores, patients answered the Wound‐QoL‐17, from which the scores of both the Wound‐QoL‐17 and the Wound‐QoL‐14 were calculated (each ranging from 0 to 4). The Wound‐QoL language versions used had been developed through a comprehensive translation process with at least two forward‐ and two backward‐translations by professional translators. Data from this study was used to validate all of the language versions applied [16, 17]. All other questions were translated in a single forward‐translation process by a professional translation office and were checked by the local investigators.

### 3.2 Development of Wound‐QoL Bands

Our methodology to develop Wound‐QoL bands was based on previous studies developing bands for other dermatological patient‐reported outcome measures, in which bands for the Dermatology Life Quality Index (DLQI) [25] and a Visual Analogue Scale for Itch [26] were developed. Similar to these studies, we defined an anchor variable, in this case the GQ, and then used descriptive statistics (absolute and relative frequency, mean, standard deviation [SD], median, mode) to present patient characteristics and Wound‐QoL scores. After this, we assessed associations between the Wound‐QoL scores and the GQ score using Spearman rank correlation. We grouped patients' Wound‐QoL scores in two different ways: (1) in steps of 0.5 and (2) in steps of 0.25. The GQ was mapped on these groups by determining its frequency distribution, mean, median and mode for each group. Based on this mapping, we developed Wound‐QoL bands. As there were Wound‐QoL scores that could be depicted to any of two adjacent bands, we developed four different sets of bands. To find the best fitting version, we calculated the weighted kappa (*κ*) coefficient of agreement for each set of bands. This procedure was conducted for both the Wound‐QoL‐17 and the Wound‐QoL‐14. The κ coefficient can range from 0 to 1 and values are rated as follows: < 0.20, poor; 0.21–0.40, fair; 0.41–0.60, moderate;0.61–0.80, good; 0.80–1.00, very good [27].

Instead of using the Wound‐QoL mean scores ranging from 0 to 4, it might be more feasible in clinical practice to use Wound‐QoL sum scores. Therefore, we translated the final set of bands into Wound‐QoL sum scores by multiplying the mean scores by 17 (for Wound‐QoL‐17) and by 14 (for Wound‐QoL‐14). As sum scores can only be integer values, decimal numbers were rounded down. Then, we analysed GQ score frequency distribution, mean, median, and mode for each Wound‐QoL sum score bands and calculated the weighted *κ* coefficient for the final set of Wound‐QoL sum score bands; missing items in the Wound‐QoL were rated as 0.

### 3.3 Analysis of Top Box Responses

Top box responses were defined as answers to items on one of the upper two response options (“quite”, “very”). We described the sum of top box responses descriptively and correlated these with the GQ score using Spearman rank correlation. Additionally, we displayed the correlation between top box responses and the GQ score in scatterplots. In order to allow an interpretation of the top box count, we developed a categorisation based on the Wound‐QoL bands. Therefore, we took the final set of Wound‐QoL sum scores and divided this by 4 (as this is the maximum rating per item), and, in case of non‐natural numbers, we rounded down. To evaluate the concordance with the Wound‐QoL scores and the GQ score, we calculated the weighted kappa (*κ*) coefficient of agreement for the top box response categorisation with the Wound‐QoL bands and the GQ score.

## 4 Results

### 4.1 Study Population

The 305 patients had a mean age of 68.5 years (SD 13.9, range: 28–96) and 52.8% (*n* = 161) were male. Patients had venous, arterial or mixed leg ulcers (49.2%, *n* = 150), diabetic foot ulcer (23.9%, *n* = 73), or other types of ulcer (19.3%, *n* = 59); for 7.5% (*n* = 23) no information on the type of wound was available. Patients were recruited in Austria (*n* = 51, 16.7%), Lithuania, Poland (each *n* = 50, 16.4%), Ukraine (*n* = 42, 13.8%), Slovakia (*n* = 41, 13.4%), the Netherlands (*n* = 37, 12.1%), Spain (*n* = 21, 6.9%), and Switzerland (*n* = 13, 4.3%).

The mean score of the Wound‐QoL‐17 was 2.02 (SD 1.01, range: 0.0–4.0, *n* = 301); the mean score of the Wound‐QoL‐14 was 2.01 (SD 1.02, range: 0.0–4.0, *n* = 301); the mean score of the GQ was 2.46 (SD 1.22, range: 0.0–4.0; *n* = 292). Both Wound‐QoL scores correlated strongly with the GQ score (Wound‐QoL‐17: *r* = 0.765, *p* < 0.001, *n* = 289; Wound‐QoL‐14: *r* = 0.751, *p* < 0.001, *n* = 289).

### 4.2 Development of Wound‐QoL Bands

For each 0.25‐step of the Wound‐QoL scores, the GQ statistics are displayed in Table 1 for Wound‐QoL‐17 and Table 2 for Wound‐QoL‐14.

**TABLE 1:** Number of patients with each 0.25‐step of the Wound‐QoL‐17 and details of corresponding GQ score.

Wound‐QoL‐17 score	GQ score								Total patients
	Not at all/rarely	A little	Moderately	Quite a lot	Very much	Mean	Median	Mode	
0–0.25	6	—	—	—	—	0.0	0.0	0.0	6
> 0.25 to 0.5	3	5	7	—	—	1.3	1.0	2.0	15
> 0.5 to 0.75	2	8	3	1	—	1.2	1.0	1.0	14
> 0.75 to 1	6	13	7	1	1	1.2	1.0	1.0	28
> 1 to 1.25	1	7	11	2	1	1.8	2.0	2.0	22
> 1.25 to 1.5	1	5	8	3	—	1.8	2.0	2.0	17
> 1.5 to 1.75	1	1	9	7	2	2.4	2.0	2.0	20
> 1.75 to 2	—	2	10	11	1	2.5	2.5	3.0	24
> 2 to 2.25	1	2	5	6	3	2.5	3.0	3.0	17
> 2.25 to 2.5	—	—	5	12	7	3.1	3.0	3.0	24
> 2.5 to 2.75	—	—	4	10	8	3.2	3.0	3.0	22
> 2.75 to 3	—	—	—	12	9	3.4	3.0	3.0	21
> 3 to 3.25	—	—	2	9	12	3.4	4.0	4.0	23
> 3.25 to 3.5	—	—	2	3	6	3.4	4.0	4.0	11
> 3.5 to 3.75	—	—	—	5	12	3.7	4.0	4.0	17
> 3.75 to 4	—	—	—	1	7	3.9	4.0	4.0	8

*Note:* Dark yellow indicates patients whose Wound‐QoL score matches the corresponding GQ score in the final Wound‐QoL bands. Light yellow indicates patients whose GQ score deviates by ±1 from the corresponding GQ score in the final Wound‐QoL bands.

**TABLE 2:** Number of patients with each 0.25‐step of the Wound‐QoL‐14 and details of corresponding GQ score.

Wound‐QoL‐14 score	GQ score								Total patients
	Not at all/rarely	A little	Moderately	Quite a lot	Very much	Mean	Median	Mode	
0–0.25	5	—	1	—	—	0.3	0.0	0.0	6
> 0.25 to 0.5	6	8	7	—	—	1.1	1.0	1.0	21
> 0.5 to 0.75	—	7	3	1	—	1.5	1.0	1.0	11
> 0.75 to 1	6	10	9	1	1	1.3	1.0	1.0	27
> 1 to 1.25	1	6	9		1	1.7	2.0	2.0	17
> 1.25 to 1.5	1	6	8	8	2	2.2	2.0	2.0	25
> 1.5 to 1.75	—	3	7	7	1	2.3	2.0	2.0	18
> 1.75 to 2	1	1	11	7	—	2.2	2.0	2.0	20
> 2 to 2.25	1	1	3	6	2	2.5	3.0	3.0	13
> 2.25 to 2.5	—	1	6	16	10	3.1	3.0	3.0	33
> 2.5 to 2.75	—	—	4	9	7	3.2	3.0	3.0	20
> 2.75 to 3	—	—	1	12	10	3.4	3.0	3.0	23
> 3 to 3.25	—	—	2	5	11	3.5	4.0	4.0	18
> 3.25 to 3.5	—	—	2	7	5	3.2	3.0	3.0	14
> 3.5 to 3.75	—	—	—	2	11	3.9	4.0	4.0	13
> 3.75 to 4	—	—	—	2	8	3.8	4.0	4.0	10

*Note:* Dark yellow indicates patients whose Wound‐QoL score matches the corresponding GQ score in the final Wound‐QoL bands. Light yellow indicates patients whose GQ score deviates by ±1 from the corresponding GQ score in the final Wound‐QoL bands.

Four different sets of Wound‐QoL bands were developed as some scores could be linked to different GQ scores. These were the same for Wound‐QoL‐17 and Wound‐QoL‐14 (Table 3).

**TABLE 3:** Weighted κ coefficient of agreement for separate possible sets of bands of the Wound‐QoL scores.

Version	Assignment of Wound‐QoL mean scores into bands					Weighted κ coefficient of agreement for Wound‐QoL‐17	Weighted κ coefficient of agreement for Wound‐QoL‐14
	Band 0	Band 1	Band 2	Band 3	Band 4		
	Not at all/rarely	A little	Moderately	Quite a lot	Very much		
V1	0–0.5	> 0.5 to 1	> 1 to 2	> 2 to 3	> 3 to 4	0.555	0.547
V2	0–1		> 1 to 2	> 2 to 3	> 3 to 4	0.542	0.532
V3	0–0.25	> 0.25 to 1	> 1 to 1.75	> 1.75 to 3	> 3 to 4	0.563	0.532
V4	0–0.25	> 0.25 to 1	> 1 to 2	> 2 to 3	> 3 to 4	0.564	0.550

**FIGURE 1:**
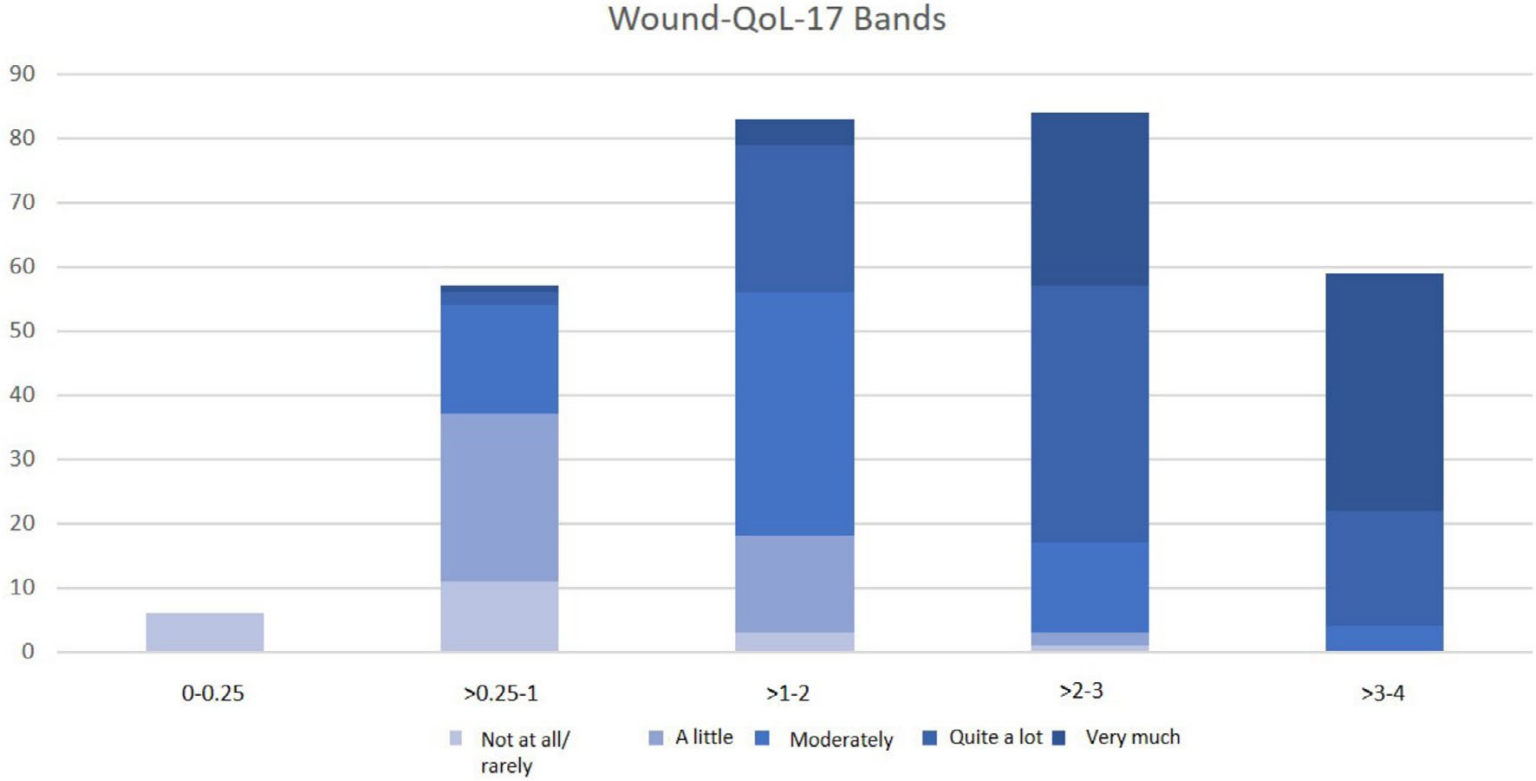
Frequency distribution of GQ scores according to Wound‐QoL‐17 bands.

**FIGURE 2:**
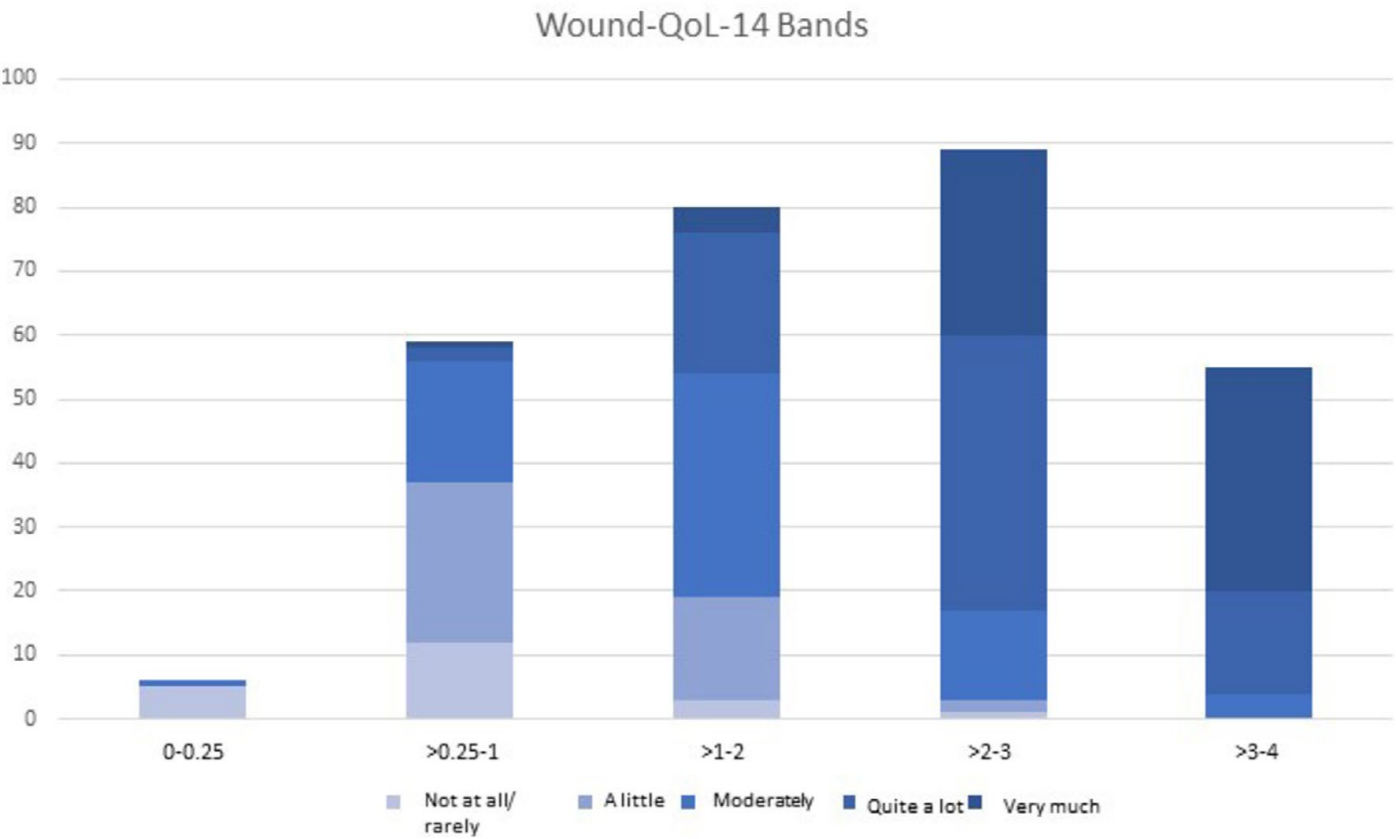
Frequency distribution of GQ scores according to Wound‐QoL‐14 bands.

Three of the sets comprised five bands each (V1, V3, and V4), each corresponding to one single GQ score. One set comprised four bands, where the first band comprised the first two GQ scores (“not at all/rarely” and “a little”). The weighted κ coefficients of agreement (Table 3) were moderate for both the Wound‐QoL‐17 (0.542 to 0.564) and the Wound‐QoL‐14 (0.532 to 0.550). The set with the highest weighted κ coefficient and, therefore, the final set of Wound‐QoL bands was 0–0.25, not at all/rarely impaired; > 0.25 to 1, a little; > 1 to 2, moderately; > 2 to 3, quite a lot; > 3 to 4, very much. The distribution of GQ scores according to the final sets of Wound‐QoL bands can be seen in Figures 1 and 2.

For the Wound‐QoL sum scores, the corresponding frequency distribution, mean, median, and mode of the GQ score can be seen in Table S1 (Wound‐QoL‐17) and Table S2 (Wound‐QoL‐14). The Wound‐QoL bands for sum scores were as follows for the Wound‐QoL‐17: 0–4, 5–17, 18–34, 35–51, 52–68 and as follows for the Wound‐QoL‐14: 0–3, 4–14,15–28, 29–42, 43–56. The weighted κ coefficient of agreement was 0.564 and 0.554, respectively.

### 4.3 Analysis of Top Box Responses

**FIGURE 3:**
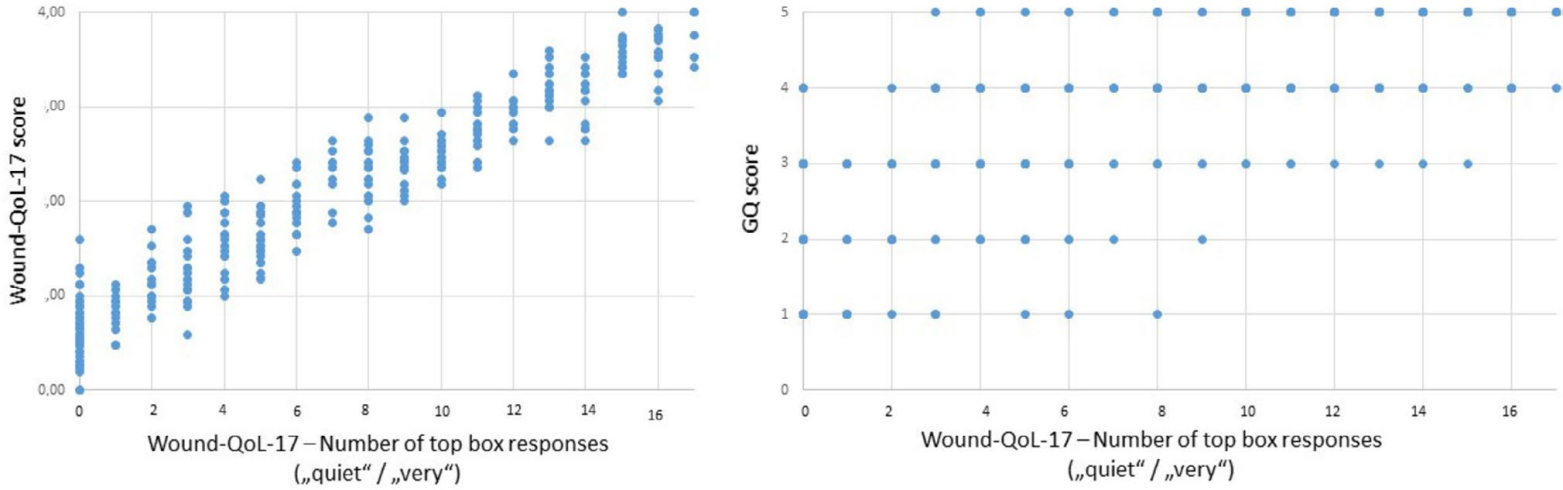
Scatterplot of number of top box responses (“quite”/“very”) in the Wound‐QoL‐17 with (a) Wound‐QoL‐17 score and (b) GQ score.

**FIGURE 4:**
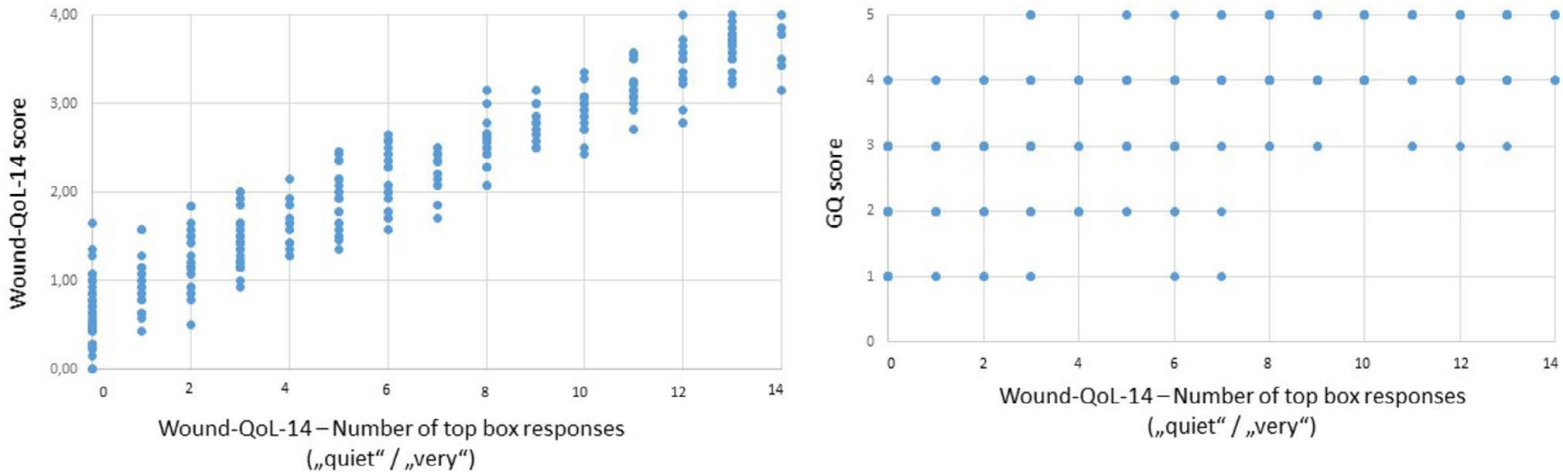
Scatterplot of number of top box responses (“quite”/“very”) in the Wound‐QoL‐14 with (a) Wound‐QoL‐14 score and (b) GQ score.

Regarding the top box responses, patients answered 7.08 (SD 5.23) items of the Wound‐QoL‐17 with one of the two upper response options, on average, and 5.78 (SD 4.37) items of the Wound‐QoL‐14. The number of top box responses correlated strongly with patients' Wound‐QoL score (Wound‐QoL‐17: *r* = 0.963, *p* < 0.001; Wound‐QoL‐14: *r* = 0.961, *p* < 0.001) as well as with patients' GQ score (Wound‐QoL‐17: *r* = 0.741, *p* < 0.001; Wound‐QoL‐14: *r* = 0.732, *p* < 0.001) showing that the more items are reported to severely impair HRQoL, the higher the patients' overall rating regarding their HRQoL impairment (Figure 3 and Figure 4).

The categorisation of the top box count based on the Wound‐QoL sum scores was as follows for the Wound‐QoL‐17: 0–1, not at all/rarely impaired; 2–4, a little; 5–8, moderately; 9–12, quite a lot; 13–17, very much. And as follows for the Wound‐QoL‐14: 0, not at all/rarely impaired; 1–3, a little; 4–7, moderately; 8–10, quite a lot; 11–14, very much.

This categorisation showed good agreement with the Wound‐QoL bands (weighted κ coefficient = 0.671 for the Wound‐QoL‐17 and 0.663 for the Wound‐QoL‐14) and moderate agreement with the GQ score (0.478 for the Wound‐QoL‐17 and 0.471 for the Wound‐QoL‐14).

## 5 Conclusion

Prior to this study, no Wound‐QoL bands had been developed. Therefore, the aim of the present analysis was to develop a categorisation of Wound‐QoL scores (i.e., Wound‐QoL bands) facilitating the interpretation of Wound‐QoL total scores.

We decided on a final set of Wound‐QoL bands that showed the best agreement with the GQ used as the anchor variable. In order to facilitate the use of the Wound‐QoL bands in different settings, we developed these bands for both the Wound‐QoL‐17 and the Wound‐QoL‐14 and also for use with sum scores.

Above this, we could show that top box responses in the Wound‐QoL questionnaire are positively correlated with overall reduced HRQoL impairments caused by the wound. Therefore, counting only the items where patients answered the two highest response options can be regarded as a good and quick approximation to the Wound‐QoL score. This approach might be time‐saving and straightforward in clinical practice.

Strengths of this study were that we have built upon a previously established methodology [25, 26] and adapted this for the Wound‐QoL. Additionally, we used data from a European study making results applicable to several language versions of the Wound‐QoL. Moreover, the use of a GQ with a verbal rating scale enabled a clear labelling of the newly developed Wound‐QoL bands.

A limitation is that the bands have been developed in a specific setting (ambulatory clinics) and generalisability might be questionable. Another limitation is that patients only completed the Wound‐QoL‐17, from which also the Wound‐QoL‐14 scores were calculated.

In future studies, patients should also complete the Wound‐QoL‐14 as a stand‐alone questionnaire instead of deriving its scores from the respective items of the Wound‐QoL‐17, as done in this study. In these studies, the GQ should also be assessed in order to test and confirm the newly developed Wound‐QoL bands.

To conclude, in this study we developed Wound‐QoL bands, which can help physicians interpret patient scores, enabling them to determine whether the HRQoL impairment of the patient can be considered not impaired at all, a little impaired, etc. Also, counting top box responses showed high agreement with the Wound‐QoL score, which supports the use of this method, especially in clinical practice. We are confident that the Wound‐QoL bands and top box count will facilitate interpretation of Wound‐QoL scores and responses in routine care as well as in research.

## Ethics Statement

The local Ethics Committee of the Medical Association of Hamburg (Ethikkommission der Ärztekammer Hamburg) approved the original study in June 2019 (PV7029). Secondary ethic votes were obtained from local ethics committees in the participating countries. For the retrospective analysis of the anonymised data in accordance with the ethical standards of the responsible committees (institutional or regional) was no further ethical approval was necessary. Data were handled according to the Helsinki Declaration of 1975, as revised in 1983.

## Conflicts of Interest

T.M.J. has no conflicts of interest. M.A. has served as consultant and/or paid speaker for and/or has received research grants and/or honoraria for consulting and/or scientific lectures for and/or got travel expenses reimbursed and/or participated in clinical trials sponsored by companies that manufacture drugs used for the treatment of wounds including 3 M, AOK Bundesverband, Bayer Healthcare, Beiersdorf, Birken, BSN, BVmed, Coloplast, DAK, Diabet concept, Mölnlycke, Smith & Nephew, Schülke & Mayr, Söring, Urgo; F.Z. has received speaker honoraria from Mölnlycke health care and Serag‐Wiessner. C.B. has received speaker honoraria and/or research grants from Amgen/Celgene, AstraZeneca, Bauerfeind, Deutsche Gesellschaft für ME/CFS, Hartmann, Lilly, Mapi Group, medi, Pfizer, Sanofi, UCB, and Urgo.

## Supporting information


**Table S1:** Number of patients with each Wound‐QoL‐17 sum score and details of corresponding GQ score.

**Table S2:** Number of patients with each Wound‐QoL‐17 sum score and details of corresponding GQ score.

## Data Availability

The data that support the findings of this study are available from the corresponding author upon reasonable request.

## References

[1] J. Dissemond , A. Bültemann , V. Gerber , B. Jäger , C. Münter , and K. Kröger , “Definitionen für die Wundbehandlung,” Hautarzt 67, no. 3 (2016): 265–266. 26769313 10.1007/s00105-016-3761-y

[2] L. Martinengo , M. Olsson , R. Bajpai , et al., “Prevalence of Chronic Wounds in the General Population: Systematic Review and Meta‐Analysis of Observational Studies,” Annals of Epidemiology 29 (2019): 8–15. 30497932 10.1016/j.annepidem.2018.10.005

[3] M. Olsson , K. Järbrink , U. Divakar , et al., “The Humanistic and Economic Burden of Chronic Wounds: A Systematic Review,” Wound Repair and Regeneration 27, no. 1 (2019): 114–125. 30362646 10.1111/wrr.12683

[4] H. Edwards , K. Finlayson , H. Skerman , et al., “Identification of Symptom Clusters in Patients With Chronic Venous Leg Ulcers,” Journal of Pain and Symptom Management 47, no. 5 (2014): 867–875. 23998779 10.1016/j.jpainsymman.2013.06.003

[5] J. A. Neil and B. A. Munjas , “Living With a Chronic Wound: The Voices of Sufferers,” Ostomy/Wound Management 46, no. 5 (2000): 28–34. 10897722

[6] C. Blome , K. Baade , E. S. Debus , P. Price , and M. Augustin , “The “Wound‐QoL”: A Short Questionnaire Measuring Quality of Life in Patients With Chronic Wounds Based on Three Established Disease‐Specific Instruments,” Wound Repair and Regeneration 22, no. 4 (2014): 504–514. 24899053 10.1111/wrr.12193

[7] T. M. Klein , V. Andrees , N. Kirsten , K. Protz , M. Augustin , and C. Blome , “Social Participation of People With Chronic Wounds: A Systematic Review,” International Wound Journal 18, no. 3 (2020): 287–311. 33314686 10.1111/iwj.13533 PMC8244007

[8] M. Bullinger , “Das Konzept der Lebensqualität in der Medizin—Entwicklung und heutiger Stellenwert,” Zeitschrift für Evidenz, Fortbildung und Qualität im Gesundheitswesen 108, no. 2–3 (2014): 97–103. 24780706 10.1016/j.zefq.2014.02.006

[9] J. Greenhalgh , K. Gooding , E. Gibbons , et al., “How Do Patient Reported Outcome Measures (PROMs) Support Clinician‐Patient Communication and Patient Care? A Realist Synthesis,” Journal of Patient‐Reported Outcomes 2 (2018): 42. 30294712 10.1186/s41687-018-0061-6 PMC6153194

[10] U.S. Department of Health and Human Services FDA Center for Drug Evaluation and Research , U.S. Department of Health and Human Services FDA Center for Biologics Evaluation and Research , and U.S. Department of Health and Human Services FDA Center for Devices and Radiological Health , “Guidance for Industry: Patient‐Reported Outcome Measures: Use in Medical Product Development to Support Labeling Claims: Draft Guidance,” Health and Quality of Life Outcomes 4 (2006): 79. 17034633 10.1186/1477-7525-4-79 PMC1629006

[11] Institut für Qualität und Wirtschaftlichkeit im Gesundheitswesen , ed., Allgemeine Methoden: Version 7.0, 7th ed. (Institut für Qualität und Wirtschaftlichkeit im Gesundheitswesen (IQWiG), 2023).

[12] M. Augustin , K. Herberger , S. J. Rustenbach , I. Schäfer , I. Zschocke , and C. Blome , “Quality of Life Evaluation in Wounds: Validation of the Freiburg Life Quality Assessment‐Wound Module, a Disease‐Specific Instrument,” International Wound Journal 7, no. 6 (2010): 493–501. 20880326 10.1111/j.1742-481X.2010.00732.x PMC7951359

[13] P. Price and K. Harding , “Cardiff Wound Impact Schedule: The Development of a Condition‐Specific Questionnaire to Assess Health‐Related Quality of Life in Patients With Chronic Wounds of the Lower Limb,” International Wound Journal 1, no. 1 (2004): 10–17. 16722893 10.1111/j.1742-481x.2004.00007.x PMC7951606

[14] M. Engelhardt , E. Spech , H. Diener , H. Faller , M. Augustin , and E. S. Debus , “Validation of the Disease‐Specific Quality of Life Wuerzburg Wound Score in Patients With Chronic Leg Ulcer,” Vasa 43, no. 5 (2014): 372–379. 25147014 10.1024/0301-1526/a000378

[15] C. C. von Stülpnagel , N. Da Silva , M. Augustin , et al., “Assessing the Quality of Life of People With Chronic Wounds by Using the Cross‐Culturally Valid and Revised Wound‐QoL Questionnaire,” Wound Repair and Regeneration 29, no. 3 (2021): 452–459. 33595907 10.1111/wrr.12901

[16] T. M. Janke , V. Kozon , S. Valiukeviciene , et al., “Assessing Health‐Related Quality of Life Using the Wound‐QoL‐17 and the Wound‐QoL‐14‐Results of the Cross‐Sectional European HAQOL Study Using Item Response Theory,” International Wound Journal 21, no. 8 (2024): e70009. 39099173 10.1111/iwj.70009 PMC11298544

[17] T. M. Janke , V. Kozon , S. Valiukeviciene , et al., “Validation of the Wound‐QoL‐17 and the Wound‐QoL‐14 in a European Sample of 305 Patients With Chronic Wounds,” International Wound Journal 21, no. 3 (2024): e14505. 38049311 10.1111/iwj.14505 PMC10898406

[18] R. Sommer , M. Augustin , C. Hampel‐Kalthoff , and C. Blome , “The Wound‐QoL Questionnaire on Quality of Life in Chronic Wounds is Highly Reliable,” Wound Repair and Regeneration 25, no. 4 (2017): 730–732. 28857375 10.1111/wrr.12578

[19] M. Augustin , E. Conde Montero , N. Zander , et al., “Validity and Feasibility of the Wound‐QoL Questionnaire on Health‐Related Quality of Life in Chronic Wounds,” Wound Repair and Regeneration 25, no. 5 (2017): 852–857. 29080332 10.1111/wrr.12583

[20] R. Sommer , C. C. von Stülpnagel , C. E. Fife , et al., “Development and Psychometric Evaluation of the U.S. English Wound‐QoL Questionnaire to Assess Health‐Related Quality of Life in People With Chronic Wounds,” Wound Repair and Regeneration 28, no. 5 (2020): 609–616. 33372379 10.1111/wrr.12837

[21] A.‐M. Fagerdahl and G. Bergström , “Translation and Validation of a Wound‐Specific, Quality‐Of‐Life Instrument (The Wound‐QoL) in a Swedish Population,” Ostomy/Wound Management 64, no. 5 (2018): 40–46. 29847310

[22] A. Gamus , H. Kaufman , E. Keren , G. Brandin , D. Peles , and G. Chodick , “Validation of “Wound QoL” Hebrew Version Disease‐Specific Questionnaire for Patients With Lower Extremity Ulcerations,” International Wound Journal 15, no. 4 (2018): 600–604. 29797545 10.1111/iwj.12903 PMC7949931

[23] E. Conde Montero , R. Sommer , M. Augustin , et al., “Validación de la versión española del cuestionario Wound‐QoL,” Actas Dermo‐Sifiliográficas 112, no. 1 (2021): 44–51. 33137321 10.1016/j.ad.2020.09.007

[24] J. Topp , C. Blome , M. Augustin , et al., “Determining the Minimal Important Difference for the Wound‐QoL Questionnaire,” Patient Preference and Adherence 15 (2021): 1571–1578. 34285475 10.2147/PPA.S315822 PMC8286720

[25] Y. Hongbo , C. L. Thomas , M. A. Harrison , M. S. Salek , and A. Y. Finlay , “Translating the Science of Quality of Life Into Practice: What Do Dermatology Life Quality Index Scores Mean?,” Journal of Investigative Dermatology 125, no. 4 (2005): 659–664. 16185263 10.1111/j.0022-202X.2005.23621.x

[26] A. Reich , E. Chatzigeorkidis , C. Zeidler , et al., “Tailoring the Cut‐Off Values of the Visual Analogue Scale and Numeric Rating Scale in Itch Assessment,” Acta Dermato‐Venereologica 97, no. 6 (2017): 759–760. 28224165 10.2340/00015555-2642

[27] D. G. Altman , Practical Statistics for Medical Research (Chapman and Hall/CRC, 1990).

