# Supplementary material for: How to Interpret Quality of Life Assessment of Patients With Chronic Wounds Using the Wound‐QoL
Source: Int Wound J. 2025 Sep 2;22(9):e70757. doi: 10.1111/iwj.70757 (PMC12405593; doi:10.1111/iwj.70757)
Supplement: Supplementary file 1 — Table S1: Number of patients with each Wound‐QoL‐17 sum score and details of corresponding GQ score. [file IWJ-22-e70757-s001.docx]

**Table S1.** Number of patients with each Wound-QoL-17 sum score and details of corresponding GQ score

| Wound-QoL-17 score (sum) | GQ score | | | | | | | | Total  patients |
| --- | --- | --- | --- | --- | --- | --- | --- | --- | --- |
|  | Not at all/rarely | A little | Moderately | Quite a lot | Very much | Mean | Median | Mode |  |
| 0 | 3 |  |  |  |  | 0.0 | 0.0 | 0.0 | 3 |
| 3 | 1 |  |  |  |  | 0.0 | 0.0 | 0.0 | 1 |
| 4 | 2 |  | 1 |  |  | 0.7 | 0.0 | 0.0 | 3 |
| 5 |  | 2 |  |  |  | 1.0 | 1.0 | 1.0 | 2 |
| 6 |  | 1 |  |  |  | 1.0 | 1.0 | 1.0 | 1 |
| 7 |  |  | 3 |  |  | 2.0 | 2.0 | 2.0 | 3 |
| 8 | 4 | 2 | 3 |  |  | 0.9 | 1.0 | 0.0 | 9 |
| 9 | 1 | 1 |  |  |  | 0.5 | 0.5 | 0.0 | 2 |
| 10 |  | 5 | 1 |  |  | 1.2 | 1.0 | 1.0 | 6 |
| 11 |  | 2 |  |  |  | 1.0 | 1.0 | 1.0 | 2 |
| 12 | 1 |  | 2 | 1 |  | 1.8 | 2.0 | 2.0 | 4 |
| 13 | 1 | 3 | 1 |  |  | 1.0 | 1.0 | 1.0 | 5 |
| 14 | 1 | 3 | 2 |  |  | 1.2 | 1.0 | 1.0 | 6 |
| 15 | 2 | 3 | 1 |  |  | 0.8 | 1.0 | 1.0 | 6 |
| 16 | 2 | 1 | 3 |  |  | 1.2 | 1.5 | 2.0 | 6 |
| 17 |  | 3 | 1 | 1 | 1 | 2.0 | 1.5 | 1.0 | 6 |
| 18 |  |  | 4 |  |  | 2.0 | 2.0 | 2.0 | 4 |
| 19 |  | 2 | 4 | 1 |  | 1.9 | 2.0 | 2.0 | 7 |
| 20 |  | 3 | 1 | 1 | 1 | 2.0 | 1.5 | 1.0 | 6 |
| 21 | 1 | 2 | 1 |  |  | 1.0 | 1.0 | 1.0 | 4 |
| 22 |  |  | 3 | 1 |  | 2.3 | 2.0 | 2.0 | 4 |
| 23 |  | 1 | 3 |  |  | 1.8 | 2.0 | 2.0 | 4 |
| 24 |  | 3 |  | 1 |  | 1.5 | 1.0 | 1.0 | 4 |
| 25 | 1 | 1 | 2 | 1 |  | 1.6 | 2.0 | 2.0 | 5 |
| 26 |  |  | 2 | 2 | 1 | 2.8 | 3.0 | 2.0 | 5 |
| 27 | 1 | 1 | 3 | 2 |  | 1.9 | 2.0 | 2.0 | 7 |
| 28 |  |  | 3 | 3 | 2 | 2.9 | 3.0 | 2.0 | 8 |
| 29 |  |  | 1 |  |  | 2.0 | 2.0 | 2.0 | 1 |
| 30 |  | 1 |  | 2 |  | 2.3 | 3.0 | 3.0 | 3 |
| 31 |  |  | 1 | 1 |  | 2.5 | 2.5 | 2.0 | 2 |
| 32 |  | 1 | 6 | 5 |  | 2.3 | 2.0 | 2.0 | 12 |
| 33 |  |  | 2 | 1 |  | 2.3 | 2.0 | 2.0 | 3 |
| 34 |  |  | 1 | 2 |  | 2.7 | 3.0 | 3.0 | 3 |
| 35 | 1 |  | 1 | 4 |  | 2.3 | 3.0 | 3.0 | 6 |
| 36 |  |  | 1 | 1 |  | 2.5 | 2.5 | 2.0 | 2 |
| 37 |  | 2 | 1 | 3 | 1 | 2.4 | 3.0 | 3.0 | 7 |
| 38 |  |  | 2 | 1 | 2 | 3.0 | 3.0 | 2.0 | 5 |
| 39 |  |  | 2 |  |  | 2.0 | 2.0 | 2.0 | 2 |
| 40 |  |  | 2 | 4 | 4 | 3.2 | 3.0 | 3.0 | 10 |
| 41 |  |  | 2 | 5 | 2 | 3.0 | 3.0 | 3.0 | 9 |
| 42 |  |  |  | 2 | 1 | 3.3 | 3.0 | 3.0 | 3 |
| 43 |  |  | 1 | 3 | 3 | 3.3 | 3.0 | 3.0 | 7 |
| 44 |  |  | 1 | 2 |  | 2.7 | 3.0 | 3.0 | 3 |
| 45 |  |  | 1 | 4 | 3 | 3.3 | 3.0 | 3.0 | 8 |
| 46 |  |  | 1 | 2 |  | 3.3 | 4.0 | 4.0 | 3 |
| 47 |  |  |  | 5 |  | 3.0 | 3.0 | 3.0 | 5 |
| 48 |  |  |  | 2 | 3 | 3.6 | 4.0 | 4.0 | 5 |
| 49 |  |  |  | 1 | 1 | 3.5 | 3.5 | 3.0 | 2 |
| 50 |  |  |  | 1 | 2 | 3.7 | 4.0 | 4.0 | 3 |
| 51 |  |  |  | 3 | 3 | 3.5 | 3.5 | 3.0 | 6 |
| 52 |  |  |  | 2 | 5 | 3.7 | 4.0 | 4.0 | 7 |
| 53 |  |  | 1 | 2 |  | 2.7 | 3.0 | 3.0 | 3 |
| 54 |  |  |  | 3 | 6 | 3.7 | 4.0 | 4.0 | 9 |
| 55 |  |  |  | 2 | 2 | 3.5 | 3.5 | 3.0 | 4 |
| 57 |  |  |  | 2 | 3 | 3.6 | 4.0 | 4.0 | 5 |
| 58 |  |  | 2 | 1 | 2 | 3.0 | 3.0 | 2.0 | 5 |
| 59 |  |  |  |  | 1 | 4.0 | 4.0 | 4.0 | 1 |
| 60 |  |  |  | 3 | 6 | 3.7 | 4.0 | 4.0 | 9 |
| 61 |  |  |  | 2 | 2 | 3.5 | 3.5 | 3.0 | 4 |
| 62 |  |  |  |  | 2 | 4.0 | 4.0 | 4.0 | 2 |
| 63 |  |  |  | 1 | 2 | 3.7 | 4.0 | 4.0 | 3 |
| 64 |  |  |  |  | 2 | 4.0 | 4.0 | 4.0 | 2 |
| 65 |  |  |  |  | 2 | 4.0 | 4.0 | 4.0 | 2 |
| 68 |  |  |  |  | 2 | 4.0 | 4.0 | 4.0 | 2 |
